# Glanzmann thrombasthenia

**DOI:** 10.1186/1750-1172-1-10

**Published:** 2006-04-06

**Authors:** Alan T Nurden

**Affiliations:** 1 IFR N°4/CRPP, Laboratoire d'Hématologie, Hôpital Cardiologique, 33604 Pessac, France

## Abstract

Glanzmann thrombasthenia (GT) is a rare autosomal recessive bleeding syndrome affecting the megakaryocyte lineage and characterized by lack of platelet aggregation. The molecular basis is linked to quantitative and/or qualitative abnormalities of **α**IIb**β**3 integrin. This receptor mediates the binding of adhesive proteins that attach aggregating platelets and ensure thrombus formation at sites of injury in blood vessels. GT is associated with clinical variability: some patients have only minimal bruising while others have frequent, severe and potentially fatal hemorrhages. The site of bleeding in GT is clearly defined: purpura, epistaxis, gingival hemorrhage, and menorrhagia are nearly constant features; gastrointestinal bleeding and hematuria are less common. In most cases, bleeding symptoms manifest rapidly after birth, even if GT is occasionally only diagnosed in later life. Diagnosis should be suspected in patients with mucocutaneous bleeding with absent platelet aggregation in response to all physiologic stimuli, and a normal platelet count and morphology. Platelet **α**IIb**β**3 deficiency or nonfunction should always be confirmed, for example by flow cytometry. In order to avoid platelet alloimmunisation, therapeutic management must include, if possible, local hemostatic procedures and/or desmopressin (DDAVP) administration. Transfusion of HLA-compatible platelet concentrates may be necessary if these measures are ineffective, or to prevent bleeding during surgery. Administration of recombinant factor VIIa is an increasingly used therapeutic alternative. GT can be a severe hemorrhagic disease, however the prognosis is excellent with careful supportive care.

## Disease name

Glanzmann thrombasthenia (GT)

## Definition/diagnostic criteria

GT is an autosomal recessive bleeding syndrome affecting the megakaryocyte lineage and characterized by a lack of platelet aggregation. It is a moderate to severe hemorrhagic disorder with mainly mucocutaneous bleeding. The molecular basis is linked to quantitative and/or qualitative abnormalities of αIIbβ3 integrin, the receptor that mediates the incorporation of platelets into an aggregate or thrombus at sites of vessel injury.

Glanzmann first described this disease in 1918 as "hereditary hemorrhagic thrombasthenia" [1]. A prolonged bleeding time and an isolated, rather than clumped, appearance of platelets on a peripheral blood smear were early diagnostic criteria. In 1956, Braunsteiner and Pakesch reviewed disorders of platelet function and described thrombasthenia as an inherited disease characterized by platelets of normal size that failed to spread onto a surface and did not support clot retraction [2]. The diagnostic features of GT including the absence of platelet aggregation as the primary feature were clearly established in 1964 by the classic report on 15 French patients by Caen *et al*. [3]. Those patients with absent platelet aggregation and absent clot retraction were subsequently termed as having type I disease; those with absent aggregation but residual clot retraction, type II disease; while variant disease was first established in 1987 (reviewed in ref [4]).

## Differential diagnosis

Platelet aggregation defects specific to adenosine diphosphate (ADP) or collagen, imply abnormalities of their primary receptors or of signaling pathways. Defects in the second wave of aggregation to ADP or in the response to collagen can imply storage pool disease and an absence of the secretory stores of ADP in dense granules. Deficiencies in the platelet response to arachidonic acid can point either to an inherited abnormality in thromboxane A2 formation or a platelet function defect temporarily acquired through aspirin ingestion. GT is the only disease in which platelet aggregation is defective to all agonists, while absent clot retraction is another frequent characteristic.

Normal ristocetin-induced platelet agglutination and normal platelet size clearly rule out the Bernard-Soulier syndrome, a disorder of platelet adhesion. Inherited thrombocytopenias are eliminated by a normal platelet count. Normal coagulation parameters rule out clotting disorders that can also affect platelet function such as congenital afibrinogenemia and von Willebrand disease. Acquired thrombasthenia must be eliminated in the absence of a family history of the disease. Platelet **α**IIb**β**3 deficiency and abnormal platelet aggregation have been reported in patients with acute promyelocytic leukemia [5]; the etiology of this acquired disorder is probably a chromosome 15–17 translocation. Although the breakpoint region on chromosome 17 is heterogeneous in acute promyelocytic leukemia, in some patients it occurs at 17q21, and this is the location of the genes for **α**IIb and **β**3 [6]. Another problem in diagnosing GT is to eliminate patients with acquired autoantibodies that block aggregation, although these patients would often be thrombocytopenic [7]. These antibodies can be detected immunologically by their binding to **α**IIb**β**3 of control platelets during incubation with the patient's serum [8].

## Etiology

### Cell biology

Megakaryocytes are found in the bone marrow and when mature, liberate large numbers of platelets into the blood circulation. In GT, platelets fail to aggregate in response to all natural agonists, including ADP, thrombin and collagen, despite their undergoing a normal shape change. Thrombasthenic platelets can also adhere to exposed subendothelial tissue and secretion from storage granules is initiated. However, the subsequent reactions of platelet spreading on the exposed surface and thrombus build-up are defective [9]. In the 1970s, Nurden and Caen demonstrated that platelets from patients with GT had selective abnormalities in their membrane glycoprotein (GP) composition [10]. This led to the recognition that the disease was provoked by specific deficiencies of GPIIb and GPIIIa. It was later established that (i) GPIIb and GPIIIa were present in the platelet membrane as a heterodimeric molecule and (ii) like **α**IIb**β**3, the complex was a member of the ubiquitous integrin family of cell surface receptors [11,12]. Significantly, GT is now generally recognized as the most frequent inherited integrin disorder.

In man, expression of the **α***IIb *gene (ITGA2B) (and therefore of **α**IIb**β**3 integrin) is restricted to cells of the megakaryocytic cell lineage. Expression of the **β***3 *gene (ITGB3) is more widespread, with the vitronectin receptor (**α**v**β**3) being expressed in many cell types, including endothelial cells, osteoblasts, smooth muscle cells, and leukocytes [12]. Despite this, patients with **β***3 *gene defects appear not to have a more severe form of the disease (discussed below). In the platelet, αvβ3 is a rare component, approximately 50 copies being found at the surface, compared to over 50,000 copies of **α**IIb**β**3 [13]. In the seconds following binding of stimuli to platelets, **α**IIb**β**3 straightens from a bent conformation and demonstrates receptor activity for fibrinogen [14]. Although fibrinogen is the predominant ligand in plasma, the role of von Willebrand factor (VWF) in conditions of high shear should be emphasized, while fibronectin, vitronectin and CD40L may also participate [15-17]. The inability to bind adhesive proteins when stimulated explains the platelet phenotype in GT. Fibrin binding to **α**IIb**β**3 allows some hemostatic function when residual integrin is present [18]. Furthermore, GT platelets appear able to attach to fibrin (independently of activated **α**IIb**β**3) under flow, suggesting the presence of an alternative platelet receptor for fibrin [19].

### Genetic basis

**Figure 1:**
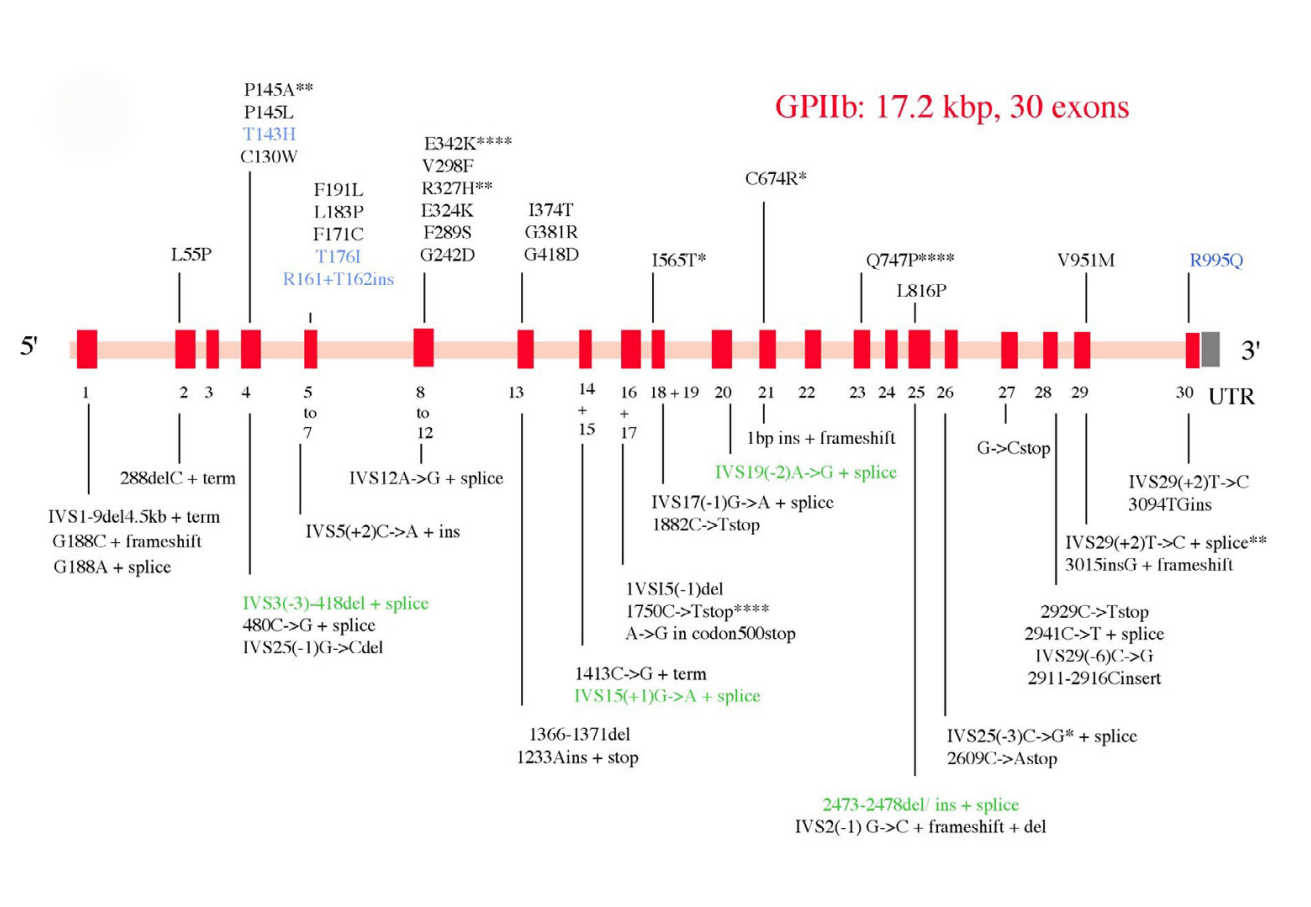
Schematic representation of the structure of the *GPIIb *(*αIIb*) gene (ITGA2B) together with a representative spectrum of the types of genetic abnormalities that give rise to Glanzmann thrombasthenia (GT). The defects responsible for variant forms of the disease are in blue type, those which are prevalent in ethnic groups are in green. Asterisks indicate the number of times that the same genetic defect has been described in apparently unrelated families. For a continually updated list of defects please consult the ISTH database . UTR: untranslated region, del = deletion, ins = insertion, inv = inversion, term = premature termination, stop = stop codon. For simplicity, the initial genetic defect is highlighted. Frameshifts and aberrant splicing are not always noted.

**Figure 2:**
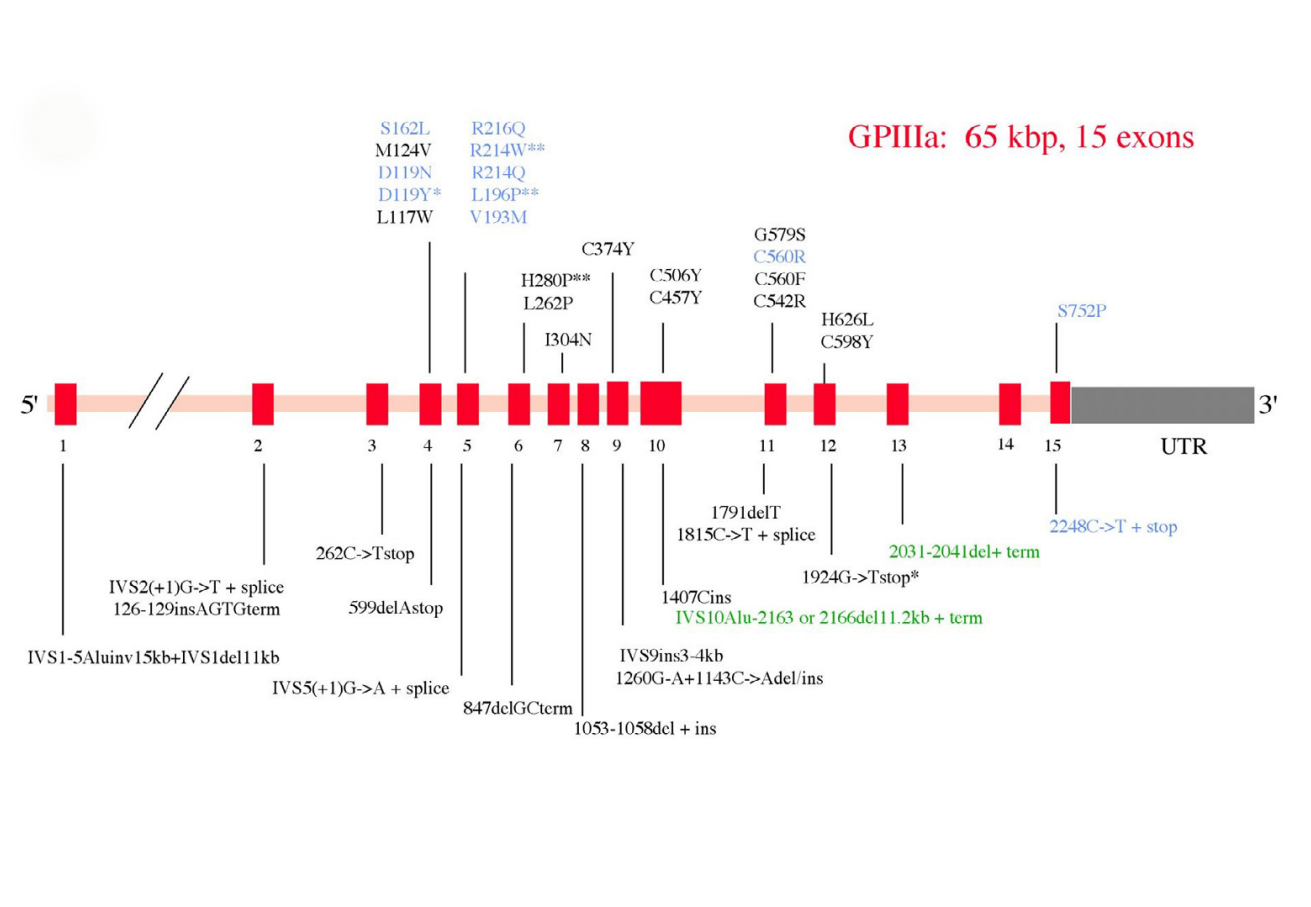
Schematic representation of the structure of the *GPIIIa *(*β3*) gene (ITGB3) together with a representative spectrum of the types of genetic abnormalities that give rise to Glanzmann thrombasthenia (GT). Note that abnormalities are abundant in both *αIIb *and *β3 *genes and that no parts of either gene appear to be exempt. For further information see the legend to Figure 1.

A continually updated database is available on the Internet : it currently contains a list of about 100 mutations giving rise to GT. The **α***IIb *and **β***3 *genes are both affected and while posttranslational defects predominate, mRNA stability can also be reduced. In brief, integrin synthesis occurs in the megakaryocytes with **α**IIb**β**3 complex formation in the endoplasmic reticulum (ER). Noncomplexed or incorrectly folded gene products fail to undergo processing in the Golgi apparatus and are rapidly degraded intracellularly [20,21]. One exception is the ability of normally synthesized **β**3 to complex with **α**v and form **α**v**β**3 (see above). Figures 1 and 2 show those mutations where supplementary information (family studies, site-directed mutagenesis) links them to the GT phenotype. Deletions and insertions, nonsense and missense mutations are common causes of GT. Splice site defects and frameshifts are also widespread. Large deletions are rare.

As shown in Figure 1, the **α***IIb *gene is composed of 30 exons. In an early and classic study, three Israeli-Arab kindreds were shown to possess a 13-bp deletion leading to a six-amino acid deletion in the **α**IIb protein [22]. The affected region, including Cys^107^, was postulated to be critical for posttranslational processing of **α**IIb. Missense mutations in exons encoding the extracellular β– propeller region of **α**IIb [23] have shown how the extracellular calcium-binding domains of **α**IIb are essential for **α**IIb**β**3 biogenesis [24-26]. Site-directed mutagenesis involving various amino acid substitutions at position 324 of **α**IIb, illustrated to what extent the GT phenotype depended on both the nature of the substituted amino acid and its replacement [26]. Mutations affecting the membrane-proximal calf-2 domain showed that while this region was not essential for complex formation in the ER, it was necessary for transport into and/or through the Golgi apparatus [21,27]. These are but a few selected examples of **α**IIb defects.

The organization of the **β***3 *gene is shown in Figure 2. It is composed of 15 exons and mutations are again widely distributed within the gene. An 11 bp deletion leading to protein termination shortly before the transmembrane domain of **β**3 was first described in six Iraqi Jews with type I disease [22]. This defect prevented normal membrane insertion of the integrin and also **α**v**β**3 expression, both in platelets and other cells. Although most **β***3 *mutations affect **α**IIb**β**3 and **α**v**β**3 expression, rare mutations allow **α**v**β**3 expression while preventing **α**IIb**β**3 processing [28].

Patients with mutations allowing **α**IIb**β**3 to be processed, but in whom integrin function is abolished, are of particular interest. In most of these patients, it is the **β***3 *gene that is affected. In brief, many of the variants have platelets with sufficient **α**IIb**β**3 to normally allow aggregation, but the activation-dependent expression of adhesive protein binding sites on the integrin does not occur [29,30]. As well as providing information on the ligand-binding pocket on the extracellular domains, variant molecules have highlighted the role of the **α**IIb and **β**3 intracellular tails in integrin signaling and even for integrin trafficking [31-33]. For some variants, clot retraction can occur even if aggregation is prevented [34]. Finally, recent studies on two patients have revealed that disruption of disulfide bridges in the **β**3 Epidermal Growth Factor (EGF) extracellular domains gives rise to a constitutively active integrin, able to spontaneously bind fibrinogen [35,36]. Here, aggregation fails to occur because of the absence of free counter receptors allowing platelet to platelet bridging.

The recent application of mutation screening on a national basis, first in Italy and then in India, has re-emphasized how a wide array of mutations can be found in GT patients within a single country. Interestingly, while 17 out of 21 candidate mutations were in the **α***IIb *gene of the Italian patients, **β**3 mutations with emphasis on exon 4 appear to characterize the Indian patients [37,38].

## Clinical description

Early reports emphasized the clinical variability of this bleeding syndrome: some patients had only minimal bruising, while others had frequent, severe and potentially fatal hemorrhages. Hemorrhagic symptoms occur only in patients homozygous for mutations causing GT; the heterozygous condition is mostly asymptomatic, even though these subjects have only a half-normal concentration of platelet **α**IIb**β**3 [4]. The sites of bleeding in GT are clearly defined: purpura, epistaxis, gingival hemorrhage, and menorrhagia are nearly constant features; gastrointestinal bleeding and hematuria are less common but can cause serious complications [39,40]. It is important to note that deep visceral hematomas, a characteristic of coagulation disorders such as hemophilia, are not usually seen. In most cases, bleeding symptoms manifest rapidly after birth, even if occasionally GT is only diagnosed later in life. Epistaxis is a common cause of severe bleeding, and is typically more severe in childhood. In general, the bleeding tendency in GT decreases with age. The rare co-existence of GT with other inherited diseases, such as mild von Willebrand disease, may accentuate the clinical severity of bleeding [27,39].

Although GT can be a severe hemorrhagic disease, the prognosis is excellent with careful supportive care. Most adult patients are in good health and their disease has a limited effect on their daily lives. Death from hemorrhage in diagnosed patients is rare unless associated with trauma or other disease (*e.g*. cancer). In contrast, families often report deceased siblings on diagnosis of GT.

### Correlation of clinical disease with the molecular platelet abnormality

Clinical observations suggest little or no correlation between the amount of residual platelet **α**IIb**β**3 and the severity of hemorrhagic disease [4,39]. Among the patients studied over many years in Paris [4], some with negligible bleeding symptoms have virtually no detectable **α**IIb**β**3, while others who have 10%–15% of the normal level of functional platelet **α**IIb**β**3 have experienced severe hemorrhage. It can be postulated that the absence of **α**v**β**3 function in vascular cells may contribute to the hemorrhagic tendency. However, even among families in whom **β**3 is undetectable, bleeding ranges from severe to moderate and sporadic. Since therapeutic inhibition of platelet **α**IIb**β**3 function prevents arterial thrombosis [41], patients are empirically protected from this disease. It has been speculated that patients with GT may also be protected from atherosclerotic disease. However, studies within ethnic groups in Israel have suggested that this is not so [42]. Patients with GT also are not protected against venous thrombosis, where plasma coagulation factors are of primary importance [43].

Mice lacking **β**3 integrins develop thickened bones because of dysfunctional osteoclasts [44]. However, upregulation of **α**2**β**1 integrin compensates for lack of **α**v**β**3 in osteoclasts from Iraqi-Jewish patients and so this feature may not translate to humans [45]. Other studies with **β**3 knockout mice show increased expression of Flk-1 on **β**3-null endothelial cells with increased vascular EGF (VEGF) signaling and enhanced angiogenesis and tumor growth [46]. How this or an observed decreased fertility translate to GT patients with **β***3 *gene defects are topics for urgent study.

## Epidemiology

A review of 177 patients with GT (of which 113 were literature reports and 64 were seen at the *Hôpital Lariboisière*, Paris) showed that 102 (58%) of the patients were female [4]. This finding may reflect the added problem of menorrhagia. The average age of the 113 patients for whom age was given was only 20. This may reflect improvements in diagnostic procedures. The frequency of consanguinity in affected families is noticeable, and GT has an increased incidence in populations in whom marriage among close relatives is an accepted custom. For example, in the review of 177 patients [4], only 12 were from the United States. In contrast, 55 patients were from Israel and Jordan, and 42 were from South India. In certain ethnic groups, such as South Indian Hindus, Iraqi Jews, French gypsies and Jordanian nomadic tribes, thrombasthenia may actually be a common hereditary hemorrhagic disorder. This has recently been borne out by a report of 382 patients in Iran [47]. For these reasons, it would be imprudent to give an estimation of worldwide prevalence.

## Diagnostic methods

Mucocutaneous bleeding with absent platelet aggregation in response to all physiologic stimuli is pathognomonic for GT, and abnormal clot retraction is rarely observed in other disorders [39]. When these two signs are associated with a normal platelet count and morphology, the diagnosis of GT is clear-cut. Use of the PFA-100 system (Dade-Behring, Miami, USA) can replace bleeding time tests for GT [48]. The PFA-100 measures the closure time when blood is passed through collagen-based filters under flow; blood from GT patients fails to plug the filter. Platelet **α**IIb**β**3 deficiency should always be confirmed in new patients, and this can be done with monoclonal antibodies and flow cytometry [24,35]. The detection of trace amounts of intracellular **α**IIb or **β**3 in a patient's platelets by western blotting [27,28], can give clues to the identity of the affected gene, while the presence of nonprocessed precursor pro-**α**IIb will suggest a block in integrin biosynthesis [25,26].

In the detailed analysis of experience gained at *Hôpital Lariboisière *in Paris, 50 out of 64 patients (78%) had type I disease, nine patients (14%) had type II disease with residual **α**IIb**β**3, and five patients (8%) were classified as variant GT [4], a subgroup diagnosed by the inability of **α**IIb**β**3 to express activation-dependent epitopes (recognized by the absence of binding of monoclonal antibodies, such as PAC-1 or FITC-fibrinogen, in flow cytometry) [29-34].

Procedures permitting the rapid screening of given mutations can only be used in ethnic groups with high consanguinity. This is the case for the Iraqi-Jewish and Arab groups in Israel and for a Manouche gypsy population in France [49-51]. Here, rapid screening procedures include allele-specific restriction enzyme analysis (ASRA) [51]. Otherwise, the patient needs to be referred to a specialist laboratory, for direct sequencing of the relevant genes.

## Carrier analysis, prenatal diagnosis and genetic counseling

Initial studies attempted to diagnose carriers of GT by measuring the number of **α**IIb**β**3 receptors on platelets. However, an occasional overlap between obligate heterozygotes and ostensibly normal donors, whose platelets had a low **α**IIb**β**3 expression, made interpretation of the results difficult. Prenatal diagnosis performed by measuring **α**IIb**β**3 on platelets isolated from cord blood has been attempted, but is accompanied by a high risk of bleeding and of spontaneous abortion [52]. It is therefore best to first identify the genetic lesion within the family in question. Carrier diagnosis can then be determined with relative ease particularly if sites are created (or lost) for restriction enzymes. Restriction digest analysis of polymerase chain reaction (PCR)-amplified fragments from DNA isolated from blood or urine was first used to screen subjects for the Iraqi-Jewish and Arab mutations [49]. The presence of these mutations could also be confirmed in prenatal diagnosis using DNA extracted from chorionic villi (discussed by French [20]). Carriers have also been detected within the French gypsy populations using ASRA methodology [51]. Finally, prenatal diagnosis in GT has been achieved using the polymorphic markers BRCA1 and THRA1 on chromosome 17 [53].

Genetic counseling can be given with the following reservations: (i) when screening is followed for a single mutation within an ethnic population, the presence of a second Glanzmann's defect may go undetected and (ii) that individuals with the same mutation may differ widely in the frequency and severity of bleeding.

## Management including treatment

Despite variations in the severity and frequency of bleeding episodes, most GT patients receive blood transfusions [39,40]. Local bleeding can be treated by local measures, such as fibrin sealants. Epistaxis and gingival bleeding are successfully controlled in most patients by nasal packing or the application of gel foam soaked in topical thrombin. Regular dental care is essential to prevent gingival bleeding. For teeth extractions, or for hemorrhage accompanying the loss of deciduous teeth, hemostasis can be significantly improved by the application of individually prepared plastic splints that provide physical support for hemostasis.

Severe menorrhagia is a frequent clinical problem and is usually associated with an excessively proliferative endometrium caused by estrogen dominance. It can be effectively treated with high doses of progesterone. Maintenance treatment with birth control pills should follow. Severe gastrointestinal bleeding is a problem in isolated cases. Iron deficiency anemia, which can develop insidiously with gingival oozing or minor menorrhagia, is a frequent problem.

Bleeding following trauma or surgical procedures can be severe and transfusions are often given by precaution or should be available on standby. Pregnancy and in particular, delivery, represent a particularly severe hemorrhagic risk. Platelet transfusions are required not only prior to delivery, but sometimes should be continued for at least a week [39]. Successful delivery by Cesarean section, with platelet transfusions, has been reported. Note that if platelet transfusions are required, the most HLA-compatible platelet concentrates must be chosen in order to avoid platelet anti-HLA alloimmunization.

The fact that most patients receive red cell and/or platelet transfusions on more than one occasion, makes the production of isoantibodies reactive with **α**IIb**β**3 likely [4,39]. Such antibodies are antigen-driven and are produced against different epitopes on the integrin [54]. They may block platelet aggregation, and lead to the rapid removal of transfused platelets by immune mechanisms. Whether a particular category of patient is more likely to form isoantibodies is as yet unknown. When present at high titer, the antibodies cause patients to become refractory to further transfusions. Antibodies have been successfully removed prior to surgery by immunoadsorption on Protein A Sepharose, although this is a complex procedure whose use is restricted to specialized centers [55]. Recently, recombinant factor VIIa (NovoSeven^®^; Novo Nordisk A/S, Malov, Denmark) has been successfully used in GT and represents an alternative approach for early cessation of bleeding, especially for patients with antibodies and/or a history of refractoriness to transfusion [56]. It is often used in association with anti-fibrinolytic agents. Thromboembolic events are a rare but potential hazard. Recombinant factor VIIa appears to enhance deposition of the **α**IIb**β**3-deficient platelets on the subendothelial matrix through their interaction with fibrin formed as a result of increased thrombin generation [19]. The stability of the newly-formed clot is increased and its permeability decreased [57]. Nevertheless, the efficacity of recombinant factor VIIa in children with GT has been questioned [58]. Rarely, in some patients, the condition has been thought to be sufficiently serious for allogeneic bone-marrow transplantation to be performed [39,59]. In the first report, donors were siblings and the transplantation was successful [59].

## Unresolved questions

Awaiting discovery, perhaps, are abnormalities of the cytoplasmic proteins now thought to regulate the activation state of **α**IIb**β**3 for adhesive proteins [60]. The observation that a series of platelet receptor gene haplotypes can markedly affect bleeding severity and bleeding times in patients with von Willebrand disease type I [61], points to the bleeding tendency in congenital disorders being governed by the score of an ensemble of risk factors. As well as gene polymorphisms affecting platelet receptors, those influencing coagulation factors and the functioning of vascular cells may also be involved. The application of proteomic and gene microarray technologies to platelet disorders such as GT may help determine whether bleeding risk in individual patients can be predicted. Although the work is at an early stage, animal models of gene therapy show that GT may be an appropriate disease for such an approach and research is progressing in this direction [62].

Finally, as national and international networks are set up, and sequencing centers become involved in genotyping, especially among ethnic groups and third world countries, healthcare of GT will improve on a worldwide basis.

## References

[B1] Glanzmann E (1918). Hereditare hamorrhagische thrombasthenie. Ein Beitrag zur Pathologie der Blutplattchen. J Kinderkranken.

[B2] Braunsteiner H, Pakesch F (1956). Thrombocytoasthenia and thrombocytopathia. Old names and new diseases. Blood.

[B3] Caen JP, Castaldi PA, Lecrec JC, Inceman S, Larrieu MJ, Probst M, Bernard J (1966). Glanzmann's thrombasthenia. I. Congenital bleeding disorders with long bleeding time and normal platelet count. Am J Med.

[B4] George JN, Caen J-P, Nurden AT (1990). Glanzmann's thrombasthenia: The spectrum of clinical disease. Blood.

[B5] Chen Y, Wu QY, Wang Z (1989). Abnormalities of platelet membrane glycoproteins in acute nonlymphoblastic leukemia [abstract]. Thromb Haemost.

[B6] Wilhide CC, Jin Y, Guo Q, Li L, Li SX, Rubin E, Bray PF (1997). The human integrin β3 gene is 63 kb and contains a 5'-UTR sequence regulating expression. Blood.

[B7] Tholouli E, Hay CRM, O'Gorman P, Makris M (2004). Acquired Glanzmann's thrombasthenia without thrombocytopenia: a severe acquired autoimmune bleeding disorder. Br J Haematol.

[B8] Macchi L, Nurden P, Marit G, Bihour C, Clofent-Sanchez G, Combrie R, Nurden AT (1998). Autoimmune thrombocytopenic purpura (AITP) and acquired thrombasthenia due to autoantibodies to GP IIb-IIIa in a patient with an unusual platelet membrane glycoprotein composition. Am J Hematol.

[B9] Patel D, Väänänen H, Jirouskova M, Hoffmann T, Bodian C, Coller BS (2003). Dynamics of GPIIb/IIIa-mediated platelet-platelet interactions in platelet adhesion/thrombus formation on collagen in vitro as revealed by videomicroscopy. Blood.

[B10] Nurden AT, Caen JP (1975). Specific roles for surface membrane glycoproteins in platelet function. Nature.

[B11] Kunicki TJ, Pidard D, Rosa JP, Nurden AT (1981). The formation of Ca2+-dependent complexes of platelet membrane glycoproteins IIb and IIIa in solution as determined by crossed immunoelectrophoresis. Blood.

[B12] Hynes RO (1992). Integrins: versatility, modulation, and signaling in cell adhesion. Cell.

[B13] Coller BS, Cheresh DA, Asch E, Seligsohn U (1991). Platelet vitronectin receptor expression differentiates Iraqi-Jewish from Arab patients with Glanzmann thrombasthenia in Israel. Blood.

[B14] Xiao T, Takagi J, Coller BS, Wang J-H, Springer TA (2004). Structural basis for allostery in integrins and binding to fibrinogen-mimetic therapeutics. Nature.

[B15] Savage B, Almus-Jacobs F, Ruggeri ZM (1998). Specific synergy of multiple substrate-receptor interactions in platelet-thrombus formation under flow. Cell.

[B16] Ni H, Denis CV, Subbarao S, Degen JL, Sato TN, Hynes RO, Wagner DD (2000). Persistence of platelet thrombus formation in arterioles of mice lacking both von Willebrand factor and fibrinogen. J Clin Invest.

[B17] Andre P, Prasad KS, Denis CV, He M, Papalia JM, Hynes RO, Phillips DR, Wagner DD (2002). CD40L stabilizes arterial thrombi by a β3 integrin-dependent mechanism. Nature Med.

[B18] Hainaud P, Brouland JP, Andre P, Simoneau G, Bal Dit Sollier C, Drouet L, Caen J, Bellucci S (2002). Dissociation between fibrinogen and fibrin interaction with platelets in patients with different subtypes of Glanzmann's thrombasthenia: studies in an ex vivo perfusion chamber model. Br J Haematol.

[B19] Lisman T, Moschatsis S, Adelmeijer J, Nieuwenhuis HK, De Groot PG (2003). Recombinant factor VIIa enhances deposition of platelets with congenital or acquired αIIbβ3 deficiency to endothelial cell matrix and collagen under flow via tissue factor-independent thrombin generation. Blood.

[B20] French DL (1998). The molecular genetics of Glanzmann's thrombasthenia. Platelets.

[B21] Rosenberg N, Yatuv R, Sobolev V, Peretz H, Zivelin A, Seligsohn U (2003). Major mutations in calf-1 and calf-2 domains of glycoprotein IIb in patients with Glanzmann thrombasthenia enable GPIIb/IIIa complex formation, but impair its transport from the endoplasmic reticulum to the Golgi apparatus. Blood.

[B22] Newman PJ, Seligsohn U, Lyman S, Coller BS (1991). The molecular genetic basis of Glanzmann thrombasthenia in the Iraqi-Jewish and Arab populations in Israel. Proc Natl Acad Sci USA.

[B23] Springer TA (1997). Folding of the N-terminal, ligand-binding region of integrin α-subunits into a β – propeller domain. Proc Natl Acad Sci USA.

[B24] Wilcox DA, Wauthier JL, Pidard D, Newman PJ (1994). A single amino acid substitution flanking the fourth calcium binding domain of αIIb prevents maturation of the αIIbβ3 complex. J Biol Chem.

[B25] Mitchell WB, Li JH, Singh F, Michelson AD, Bussel J, Coller BS, French DL (2003). Two novel mutations in the αIIb calcium-binding domains identify hydrophobic regions essential for αIIbβ3 biogenesis. Blood.

[B26] Milet-Marsal S, Breillat C, Peyruchaud O, Nurden P, Combrie R, Nurden AT, Bourre F (2002). Analysis of the amino acid requirement for a normal αIIbβ3 maturation at αIIbGlu324 commonly mutated in Glanzmann thrombasthenia. Thromb Haemost.

[B27] Nurden AT, Breillat C, Jacquelin B, Combrie R, Freedman J, Blanchette VS, Schmugge M, Rand ML (2004). Triple heterozygosity in the integrin αIIb subunit in a patient with Glanzmann thrombasthenia. J Thromb Haemost.

[B28] Tadokoro S, Tomiyama Y, Honda S, Kashiwagi H, Kosugi S, Shiraga M, Kiyoi T, Kurata Y, Matsuzawa Y (2002). Missense mutations in the β3 subunit have a different impact on the expression and function between αIIbβ3 and αvβ3. Blood.

[B29] Loftus JC, O'Toole TE, Plow EF, Glass A, Frelinger AL, Ginsberg MH (1990). A β3 integrin mutation abolishes ligand binding and alters divalent cation-dependent conformation. Science.

[B30] Lanza F, Stierle A, Fournier D, Morales M, Andre G, Nurden AT, Cazenave JP (1992). A new variant of Glanzmann's thrombasthenia (Strasbourg I). Platelets with functionally defective glycoprotein IIb-IIIa complexes and a glycoprotein IIIa 214Arg->Trp mutation. J Clin Invest.

[B31] Chen YP, Djaffar I, Pidard D, Steiner B, Cieutat AM, Caen JP, Rosa JP (1992). Ser752->Pro mutation in the cytoplasmic domain of integrin β3 subunit and defective activation of platelet integrin αIIbβ3 (glycoprotein IIb-IIIa) in a variant of Glanzmann's thrombasthenia. Proc Natl Acad Sci USA.

[B32] Wang R, Shattil SJ, Ambruso DR, Newman PJ (1997). Truncation of the cytoplasmic domain of β3 in a variant form of Glanzmann thrombasthenia abrogates signaling through the integrin αIIbβ3 complex. J Clin Invest.

[B33] Peyruchaud O, Nurden AT, Milet S, Macchi L, Pannochia A, Bray PF, Kieffer N, Bourre F (1998). R to Q aminoacid substitution in the GFFKR sequence of the cytoplasmic domain of the integrin αIIb subunit in a patient with a Glanzmann's thrombasthenia-like syndrome. Blood.

[B34] Kiyoi T, Tomiyama Y, Honda S, Tadokoro S, Arai M, Kashiwagi H, Kosugi S, Kato H, Kurata Y, Matsuzawa Y (2003). A naturally occurring Tyr143HisαIIb mutation abolishes αIIbβ3 function for soluble ligands but retains its ability for mediating cell adhesion and clot retraction: comparison with other mutations causing ligand binding defects. Blood.

[B35] Ruiz C, Liu CY, Sun QH, Sigaud-Fiks M, Fressinaud E, Muller JY, Nurden P, Nurden AT, Newman PJ, Valentin N (2001). A point mutation in the cysteine-rich domain of glycoprotein (GP) IIIa results in the expression of a GPIIb-IIIa (αIIbβ3) integrin receptor locked in a high affinity state and a Glanzmann thrombasthenia-like phenotype. Blood.

[B36] Chen P, Melchior C, Brons NH, Schlegel N, Caen J, Kieffer N (2001). Probing conformational changes in the I-domain and the cysteine-rich repeat of human β3 integrins following disulfide bond disruption by cysteine mutations: identification of cysteine 598 involved in αIIbβ3 activation. J Biol Chem.

[B37] D'Andrea G, Colaizzo D, Vecchione G, Grandone E, Di Minno G, Margaglione M (2002). GLAnzmann's Thrombasthenia Italian Team (GLATIT): Glanzmann's thrombasthenia: Identification of 19 new mutations in 30 patients. Thromb Haemost.

[B38] Nair S, Ghosh K, Shetty S, Mohanty D (2005). Mutations in GPIIIa molecule as a cause for Glanzmann thrombasthenia in Indian patients. J Thromb Haemost.

[B39] Nurden AT, George JN, RW Colman, VJ Marder, AW Clowes, JN George, SZ Goldhaber (2005). Inherited abnormalities of the platelet membrane: Glanzmann thrombasthenia, Bernard-Soulier syndrome, and other disorders. "Hemostasis and Thrombosis, Basic Principles and Clinical Practice".

[B40] Bellucci S, Caen J (2002). Molecular basis of Glanzmann's thrombasthenia and current strategies in treatment. Blood Rev.

[B41] Nurden AT, Poujol C, Durrieu-Jais C, Nurden P (1999). Platelet glycoprotein IIb/IIIa inhibitors: Basic and clinical aspects. Arterioscler Thromb Vasc Biol.

[B42] Shpilberg O, Rabi I, Schiller K, Walden R, Harats D, Tyrrell KS, Coller B, Seligsohn U (2002). Patients with Glanzmann thrombasthenia lacking platelet glycoprotein αIIbβ3 (GPIIb/IIIa) and αvβ3 receptors are not protected from atherosclerosis. Circulation.

[B43] Ten Cate H, Brandjes DPM, Smits PHM, Van Mourik JA (2003). The role of platelets in venous thrombosis: a patient with Glanzmann's thrombasthenia and a factor V Leiden mutation suffering from deep venous thrombosis. J Thromb Haemost.

[B44] McHugh KP, Hodivala-Dilke K, Zheng MH, Namba N, Lam J, Novack D, Feng X, Ross FP, Hynes RO, Teitelbaum SL (2000). Mice lacking β3 integrins are osteosclerotic because of dysfunctional osteoclasts. J Clin Invest.

[B45] Horton MA, Massey HM, Rosenberg N, Nicholls B, Seligsohn U, Flanaghan AM (2003). Upregulation of osteoclast α2β1 integrin compensates for lack of αvβ3 vitronectin receptor in Iraqi-Jewish-type Glanzmann thrombasthenia. Br J Haematol.

[B46] Reynolds AR, Reynolds LE, Nagel TE, Lively JC, Robinson SD, Hicklin DJ, Bodary SC, Hodivala-Dilke KM (2004). Elevated Flk1 (vascular endothelial growth factor receptor 2) signaling mediates enhanced angiogenesis in β3-integrin-deficient mice. Cancer Res.

[B47] Toogeh G, Sharifian R, Lak M, Safaee R, Artoni A, Peyvandi F (2004). Presentation and pattern of symptoms in 382 patients with Glanzmann thrombasthenia in Iran. Am J Hematol.

[B48] Buyukasik Y, Karakus S, Goker H, Haznedaroglu IC, Ozatli D, Sayinalp N, Ozcebe OI, Dundar SV, Kirazli S (2002). Rational use of the PFA-100 device for screening of platelet function disorders and von Willebrand disease. Blood Coag Fibrinolysis.

[B49] Peretz H, Seligsohn U, Zwang E, Coller BS, Newman PJ (1991). Detection of the Glanzmann's thrombasthenia mutations in Arab and Iraqi-Jewish patients by polymerase chain reaction and restriction analysis of blood and urine samples. Thromb Haemost.

[B50] Schlegel N, Gayet O, Morel-Kopp MC, Wyler B, Hurtaud-Roux MF, Kaplan C, McGregor J (1986). The molecular genetic basis of Glanzmann's thrombasthenia in a gypsy population in France: Identification of a new mutation on the αIIb gene. Blood.

[B51] Ruan J, Peyruchaud O, Nurden P, Cazes E, Combrie R, Bourre F, Nurden AT (1998). Family screening for a Glanzmann's thrombasthenia mutation using PCR-SSCP. Platelets.

[B52] Seligsohn U, Mibashan RS, Rodeck CH, Nicolaides KH, Millar DS, Coller BS (1985). Prenatal diagnosis in Glanzmann's thrombasthenia. Lancet.

[B53] French DL, Coller BS, Usher S, Berkowitz R, Eng C, Seligsohn U, Peretz H (1998). Prenatal diagnosis of Glanzmann thrombasthenia using the polymorphic markers BRCA1 and THRAI on chromosome 17. Br J Haematol.

[B54] Jacobin MJ, Laroche-Traineau J, Little M, Keller A, Peter K, Welschof M, Nurden A, Clofent-Sanchez G (2002). Human IgG monoclonal anti-αIIbβ3-binding fragments derived from immunized donors using phage display. J Immunol.

[B55] Martin I, Kriaa F, Proulle V, Guillet B, Kaplan C, D'Oiron R, Debre M, Fressinaud E, Laurian Y, Tchernia G, Charpentier B, Lambert T, Dreyfus M (2002). Protein A Sepharose immunoadsorption can restore the efficacy of platelet concentrates in most patients with Glanzmann's thrombasthenia and anti-glycoprotein IIb-IIIa antibodies. Br J Haematol.

[B56] Poon MC, D'Oiron R, Von Depka M, Khair K, Negrier C, Karafoulidou A, Huth-Kuehne A, Morfini M (2004). International Data Collection on Recombinant Factor VIIa and Congenital Platelet Disorders Study Group: Prophylactic and therapeutic recombinant factor VIIa administration to patients with Glanzmann's thrombasthenia: results of an international survey. J Thromb Haemost.

[B57] He S, Jacobsson Ekman G, Hedner U (2005). The effect of platelets on fibrin gel structure formed in the presence of recombinant factor VIIa in hemophilia plasma and in plasma from a patient with Glanzmann thrombasthenia. J Thromb Haemost.

[B58] Almeida AM, Khair K, Hann I, Liesner RI (2003). The use of recombinant factor VIIa in children with inherited platelet function disorders. Br J Haematol.

[B59] Bellucci S, Devergie A, Gluckman E, Tobelem G, Lethielleux P, Benbunan M, Schaison G, Boiron M (1985). Complete correction of Glanzmann's thrombasthenia by allogeneic bone-marrow transplantation. Br J Haematol.

[B60] Shattil SJ, Newman PJ (2004). Integrins: dynamic scaffolds for adhesion and signaling in platelets. Blood.

[B61] Kunicki TJ, Federici AB, Salomon DR, Koziol JA, Head SR, Mondala TS, Chismar JD, Baronciani L, Canciani MT, Peake IR (2004). An association of candidate gene haplotypes and bleeding severity in von Willebrand disease (VWD) type I pedigrees. Blood.

[B62] Wilcox DA, White GC (2003). Gene therapy for platelet disorders: studies with Glanzmann's thrombasthenia. J Thromb Haemost.

